# Tension and Shear Behaviour of Basalt Fiber Bio-Composites with Digital Image Correlation and Acoustic Emission Monitoring

**DOI:** 10.3390/polym16101331

**Published:** 2024-05-09

**Authors:** Tomaž Kek, Roman Šturm, Zoran Bergant

**Affiliations:** Faculty of Mechanical Engineering, University of Ljubljana, Aškerčeva 6, 1000 Ljubljana, Slovenia; tomaz.kek@fs.uni-lj.si (T.K.); roman.sturm@fs.uni-lj.si (R.Š.)

**Keywords:** acoustic emission, basalt fiber composite, digital image correlation, damage mechanisms

## Abstract

This research investigates the mechanical behavior and damage evolution in cross-ply basalt fiber composites subjected to different loading modes. A modified Arcan rig for simultaneous acoustic emission (AE) monitoring was designed and manufactured to apply quasi-isotropic shear, combined tensile and shear loading, and pure tensile loading on specimens with a central notch. Digital image correlation (DIC) was applied for high-resolution strain measurements. The measured failure strengths of the bio-composite specimens under different loading angles are presented. The different competing failure mechanisms that contribute to the local reduction in stress concentration are described. Different damage mechanisms trigger elastic waves in the composite, with distinct AE signatures that closely follow the sequence of fracture mechanisms. AE monitoring is employed to capture signals associated with structural damage initiation and progression. The characteristic parameters of AE signals are correlated with crack modes and damage mechanisms. The evolution of AE parameters during the peak load transition is presented, which enables the timely AE detection of the maximum load transition. The combination of DIC and AE monitoring improves understanding of the mechanical response and failure mechanisms in cross-ply basalt fiber composites, offering valuable insights for possible performance monitoring and structural reliability in diverse engineering applications.

## 1. Introduction

High-performance fiber-reinforced polymers (FRPs) stand out as pivotal composite materials across diverse structural applications in industries like aerospace, marine, automotive, and sports [1,2,3,4,5]. Basalt fibers are relatively novel reinforcement materials in composite fabrication [6,7]. They are natural fibers formed through the melting of volcanic rock to about 1500 °C, then extruded through small nozzles to produce continuous filaments of basalt fiber [8]. Basalt is heated only once; further processing is carried out with cold technologies with low energy costs. Although they are not biodegradable, they are still considered natural fibers because the raw material is found in nature [7,9]. They contain no carcinogens or other health hazards [10]. They exhibit high resistance to various chemicals [11,12], excellent performance at elevated temperatures, significant damping of vibration and sound [13], low hygroscopicity, and competitive pricing [14,15]. Basalt fibers have greater tensile strength than E-fiberglass, a greater fracture load than carbon fibers, and good resistance to the chemical impact after the impact load [14,16].

Understanding the material properties of FRPs is crucial for accurately predicting how these composites will behave under stress, a process essential during the product development stage. This involves conducting material characterization, which is especially critical due to the anisotropic nature of composite materials, meaning their properties vary across different principal material directions and must be assessed as per specific testing standards. The resistance of composite materials to shear stress is notably compromised when exposed to concurrent tensile and shear stresses. This decline in performance is evident within the failure envelope’s first quadrant for shear and normal stress, as illustrated by advanced failure theories like Puck’s [17] and Cuntze’s [18].

At the macroscopic scale the ultimate failure and degradation pattern of a FRP appears to be complex, even in the case of simple macroscopic loading [19]. Every complex state of degradation is a superimposition of individual mechanisms that can also be classified by the micro (scale of fiber diameter) and meso scales (scale of ply). Besides the tensional failure in the composites, there is also literature on the compressive failure of FRP [20,21]. Damage types at FRP can be also classified as intralaminar or interlaminar damage mechanisms. Intralaminar damage mechanisms that are manly induced by in-plane loading are diffuse damage and transverse cracking [22]. Several studies have focused on transverse cracking [23,24] and diffuse damage [25,26]. Diffuse damage is connected to the fiber/matrix interface debonding and microcracks in the matrix. It is mainly induced by in-plane shear loading with homogeneous distribution across the thickness of the elementary meso scale plies. On the other hand, the transverse cracking mechanism is triggered by significant tensile stresses and relatively low shear stresses [27]. Diffuse damage results in the substantial reduction in the stiffness under shear loading [26]. The matrix microcracking phenomenon is different from the transverse cracking damage mechanism that can be described as a percolated crack through the whole thickness of the elementary ply, and is defined as a meso scale crack. Transverse cracking in fiber direction loading initiates early on and can be a predominant form of damage [22]. They appear much latter in shear loading after multiple debonded surfaces merge together [25].

Acoustic emission testing has proven to be a suitable non-destructive method for the damage characterisation of FRP composites and other engineering materials. It can be used as a standalone technique or in combination with other NDT methods [28,29,30,31,32,33,34,35,36,37,38]. In the literature, some characteristic parameters of acoustic emission (AE) signals are used to describe crack modes and damage mechanisms [28,39,40]. One of these informative parameters for the description of crack modes and the characterisation of damage is the average frequency (AF) (MHz), an estimate of the fundamental frequency content in the waveform. It is the ratio of threshold crossings over the duration of the signal. The parameter RA is the ratio of the rise time (RT) to the peak amplitude (A) of the signal and is measured in s/V. The signal energy (E), which can be associated with the energy release of various phenomena in stressed material, is defined as the integral of the square of the signal (V^2^s) (often expressed in dimensionless form). AE signals provide a tangible insight to describe crack modes and damage mechanisms across a whole cross-section of the sample over time and load. Additionally, digital image correlation (DIC) enables the accurate visualization of the surface displacement and strain field. The advantage is that the complementary information provided by DIC makes assumptions about the interpretation of AE trends more informative, since AE parameters are strongly related to visible strain field changes.

However, there is scarce information on the correlation of detected AE signals to biaxial stresses in FRP composites. The possibility of identifying the different acoustic responses of the same material under different loading stress combinations can possibly be used for structural health monitoring in real composite structures. Also, the AE can be used as a predictive tool for evaluation the tensile–shear loading combination and final material failure prediction. The fracture mechanisms of notched CFRP laminates for tension and compression are well documented in the accessible literature. There are less data for shear and biaxial loading, but the equivalent work for bio-composites is lacking. This paper is aiming to address this shortfall with the simultaneous AE sensing. A modified Arcan rig was designed and manufactured to apply quasi-isotropic in-plane shear, combined tensile and shear loading and pure tensile loading on quasi-isotropic specimens with a central notch. The different competing failure mechanisms that contribute to the local reduction in stress concentration are described. A modified Arcan rig has been made that allows the attachment of AE sensors via a screw connection for the purpose of accompanying AE monitoring on butterfly samples. Different AE signal indices are proposed for the classification of the diffuse damage and transverse cracking failure mechanisms during specimen loadings before and after reaching peak loading. DIC was used for the measurement of the strain field and as a supporting tool for damage indications. AE signals relate to different stress combinations and different failure mechanisms in composites under loading.

## 2. Materials and Methods

### 2.1. Specimen Manufacturing

The composites were manufactured using Basalt BAS BI 620 (+/−45°) bi-axial fabrics, weighing 310 g/m^2^ per layer (BASALTEX NV, Wevelgem, Belgium). The fibers were pre-treated with sizing, making them compatible for the production of epoxy matrix composites. For production, vacuum infusion (VI) or vacuum-assisted resin transfer molding (VARTM) methods were employed to create flat plates. Dry fabric materials are laid into the mold, and the vacuum is applied before resin is introduced. Once a complete vacuum is achieved, resin is pulled into the laminate using the vacuum. Vacuum infusion enables very high resin-to-fiber ratios, and improved mechanical properties of the laminates.

To construct each plate, 8 layers measuring 620 × 300 mm were cut and stacked on a polished surface made of aluminum alloy 5083. The mold, with dimensions of 700 × 380 × 20 mm from Plancast (AlCu Kamnik, Slovenia), was prepped by coating its surface with Formula 5 Release Wax Film (Rexco, Conyers, GA, USA).

During assembly, inlet and outlet tubes were positioned at both ends of the plate’s longer square side. A peel ply fabric was placed atop the dry flax fabric, followed by a vacuum polyethylene infusion mesh Diatex OM 70 (R&G GmbH, Waldenbuch, Germany). A vacuum sealing tape of 2.5 × 12 × 15 mm (R&G GmbH, Waldenbuch, Germany), was used to seal the gap between the film and the mold, as well as around the inlet and outlet tubes. The vacuum bagging film PO120 (R&G GmbH, Waldenbuch, Germany) was applied over the sheet and properly sealed. The inlet tube was connected to a vacuum gauge, while the outlet tube was linked to the Value V-i240SV vacuum pump (Value, Poland). The mold was positioned on a flat aluminum heater to maintain a temperature of 40 °C, facilitating quicker resin flow. The Greenpoxy 56 and hardener SD 7561 (Sicomin West, Pluguffan, France) were used to impregnate basalt fibers. The resin was produced with a high carbon content, originating from the plant. The molecular structure of Greenpoxy 56 was almost 51% bio-based. The glass transition temperature of the resin after curing was between 78–85 °C. Subsequently, the plate was consolidated in an autoclave for 8 h at 80 °C and a pressure of 7 bars. The supplementary autoclaving of vacuum-infused plates resulted in the production of porosity-free basalt composite laminate. The autoclave was equipped with a heating and air recirculation system that enables homogeneous temperature in the chamber. The temperature of the laminate during consolidation was controlled with type K thermocouples. 

**Figure 1:**
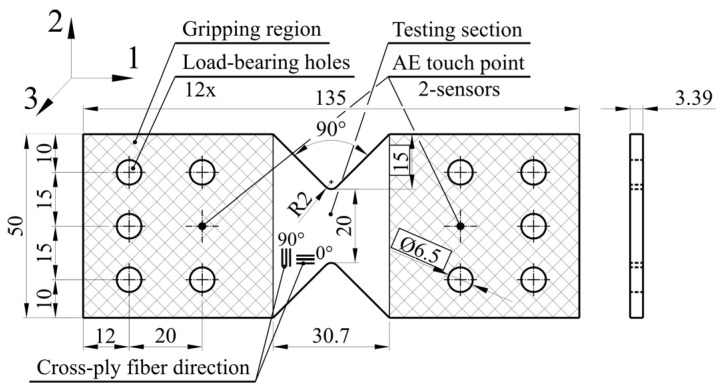
Specimen dimensions and AE coupling locations.

Arcan specimens were cut with custom 3 axis waterjet system (Omax, Richmond, KY, USA). To produce cross-ply composites, Arcan samples were diagonally cut at 45° to obtain a total of 15 specimens with [0/90]_4s_ layup. The fiber volume fraction was calculated as follows:

(1) $$\begin{array}{c} V_{f}=\frac{\rho_{m}\;\cdot \;w_{f}}{\rho_{m}\;\cdot \;w_{f}\;+\;\rho_{f}\;\cdot \;w_{m}} \end{array}$$

where $\rho_{m}$ is the matrix density, $\rho_{f}$ is the fiber density, $w_{m}$ is the matrix mass and $w_{f}$ is the fiber mass. The fiber volume fraction of vacuum-infused basalt composite is 0.55, which means that 55% of the volume fraction is fibers and 45% is epoxy resin. The specimen’s gross dimensions are 135 mm × 50 mm, a thickness of 3.48 mm (±0.02) and a test section with V-notch with an area of 20 × 3.39 mm, Figure 1. The specimens were secured between Arcan clamps using 9 M6 bolts, wherein 2 sites were left for an AE touch point. To accommodate the inner bolts, 10 holes with a diameter of 6.5 mm were drilled through the specimen. This clamping method ensured that the load was distributed not only through friction, but also through the bolts, to prevent sliding.

### 2.2. Acoustic Emission Monitoring and DIC 

On each side of the sample, threaded AE sensors were fixed in the Arcan plate. A broadband piezoelectric AE sensor Steveco KRNBB-PCP12042 (University of California, Steven D. Glaser, Berkeley, CA, USA) with a frequency bandwidth of 20 kHz to 1000 kHz and a resonance piezoelectric AE sensor VS370-A1 (Vallen Systeme, Wolfratshausen, Germany) with a frequency range of 170 to 590 kHz were attached. We used silicon grease for acoustic coupling. Attaching the sensors with a thread allowed for the good coupling of the sensors with the surface of the samples, and enabled the acquisition of AE signals, revealing the FRP damage mechanisms during loading. The layout of the test enables shorter samples to avoid excessive bending and, as a result, provides limited space with a short distance between Arcan rigs. This space limitation with the use of butterfly specimens reduces the suitability of attaching sensors with magnetic holders or clamps. The linear localisation method is used to filter signals, to deal with acoustic events in the active area of the specimen, eliminating mechanical noise from the grips. For capturing AE hits, we used the AMSY-5 device manufactured by Vallen. The sensors were connected to the device via AEP5 preamplifiers that have a 34 dB gain. The signal acquisition threshold was 40 dB, and a sampling rate of 5 MHz was used. 

Digital image correlation (DIC) analysis of displacement and strains was performed on a Q-400 DIC system (DANTEC Dynamics GmbH, Ulm, Germany). The system consists of two 2 Mpx digital cameras with 35 mm focal length lenses and a synchronization time box. Speckle patterns had a matt white spray paint base coat with black speckles which were applied using an airbrush. Stereo DIC technique was applied. The digital image software ISTRA 4D (ver 4.6) was used to analyze the results. The optical microscope Keyence VHX-6000 3D was used to obtain cross-sectional images of specimens. It was also used for 3D topography measurement based on composite image by compiling images at different focal planes. 

### 2.3. Modified Arcan Specimens 

**Figure 2:**
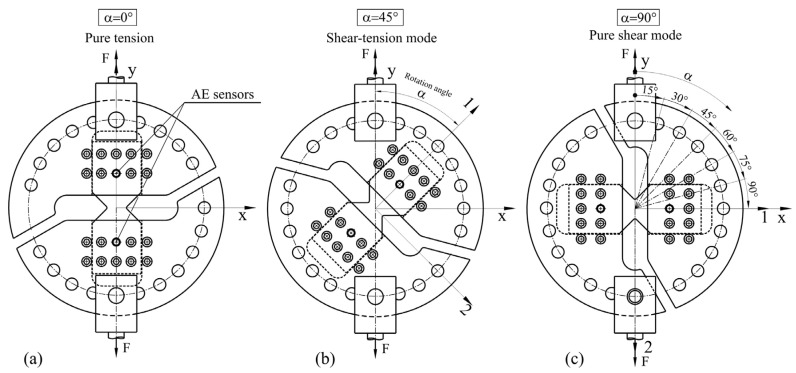
The modified Arcan rig (**a**) pure tension; (**b**) shear-tension mode; (**c**) simple shear mode.

The specimens were gripped to the modified Arcan grip using a total of 18 M6 bolts, with each bolt possessing a torque of 20 Nm. The assembly was then loaded using tensile testing machine with 1 mm/min in tension, shear, and combined tension and shear by varying the loading angle α, as defined in Figure 2. The modified Arcan rig enabled the application of loads at loading angle intervals of 15°. When α equals 90°, the loading is simple shear. An intermediate angle α between 0° and 90° results in combination of tension and shear. If the applied load is F, then the tensile component of load equals Fcosα and the shear component equals Fsinα. 

## 3. Results

### 3.1. Mechanical Properties

**Figure 3:**
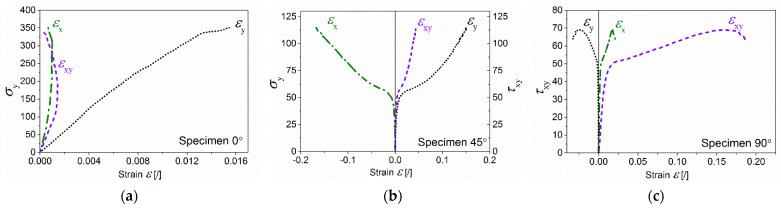
Stress–strain relationship with normal and shear stresses as a function of normal strains εx, εy, and shear strains εxy for (**a**) pure tension (0° loading); (**b**) 45° loading; and (**c**) simple shear (90° loading).

A series of biaxial loading tests were conducted, with an angle step of 45 degrees. Shear and normal strains were extracted via the DIC system at the gauge area of each specimen, Figure 3 shows the evolution of the typical stress–strain relationships during monotonic loading for the basalt bio-composites under different loading angles. Measured peak loads of 5 specimens were used for calculation of net section strength and are summarized in Table 1. A combined stress state with biaxial loading affects the lower load capacity of the material. The bearing capacity of the composite decreases with increasing shear stress, which leads to earlier failure at lower failure forces. The normal stress in pure tension reaches the highest value in an average of 345 MPa at 1.54% of ε_y_. The x strain component ε_x_ and shear strain ε_xy_ remain positive but at low values. The 45° specimen is in the biaxial state of stress. The ε_y_ strain is positive; therefore, material is being extended in the y-direction, and ε_x_ strain component has a negative sign indicating a compression deformation in the x-direction. In Figure 3c, a simple shear stress–strain relationship is depicted. In this case, the maximum shear stress reaches the average value of 69 MPa; the shear strain component ε_xy_ is the highest among all tested specimens, and reaches 18.2% at maximum shear stress. The ε_x,_ε_y_ are close to zero at the linear part, but in the plastic region, they increase due to a non-linear deformation.

### 3.2. Damage Mechanisms and Acoustic Emission Signals

**Figure 4:**
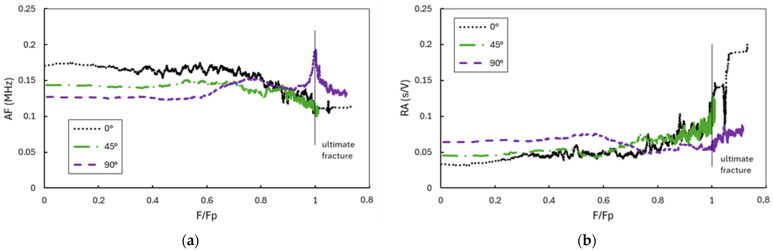
Evolution of AE parameters (**a**) AF and (**b**) RA with normalized force change.

Acoustic emission testing was applied to monitor the acoustic response of the loaded composite specimens throughout the loading sequence. The values of the AE hits parameters are highly variable during the progression of fracture damage evolution. To improve the presentation of the selective informative AE parameters, a moving average of 280 consecutive hits is used to present the captured AE signals. With the aim of improving the comparison of different loading modes, the display of AE parameters according to the normalized loading force is used (Figure 4). In the early phase of loading, higher values of AF are noticeable during pure tensile loading, which decrease with decreasing tensile stresses and increasing shear stress. In simple shear, the AF is the lowest, indicating the shear crack mode. The opposite trend can be observed with the RA parameter, with higher values detected at shear and lower values towards the pure tension. This correlates with the JCMS-IIIB5706 [41] code definition that classifies the detected tensile and shear crack mode signals according to the AF and RA value. With increasing loading force above 0.6 F_p_, AF in simple shear increases on average and approaches the values for tensile loading, which correlates with the occurrence of 1 axis fiber rotation in the FRP specimen.

**Figure 5:**
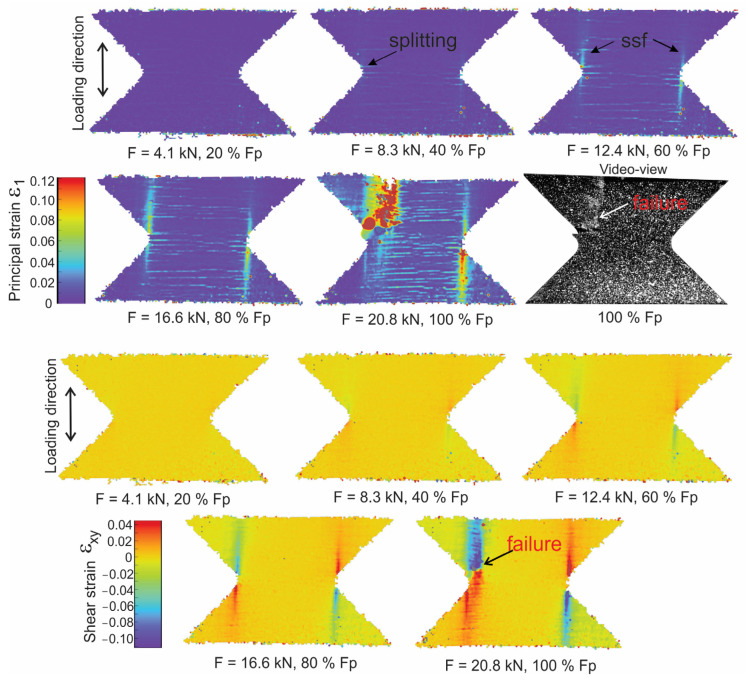
The 0° loading DIC strain field for principal strain ε_1_ and shear strain ε_xy_ (ssf—symmetrical split formation in the direction of the load).

**Figure 6:**
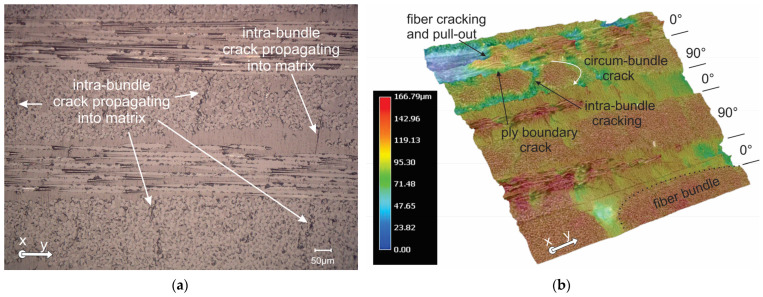
The 0° loading composite: (**a**) after loading to 0.85·F_p_ at 200X; and (**b**) after reaching F_p_.

For the applied loading modes (tenson, shear and combination), cracks in the transverse tows regarding the 1 axis are the first stress-induced cracks to be detected, as shown on Figure 5. Splitting that can be predominantly caused by intra-bundle cracks, and some matrix microcracking along the fiber-matrix interface, causes a local weakening of the composite. During the pure tension, the symmetrical split formations in the direction of the load arising from the notch area are also evident as vertical lines in strain field on Figure 5. These splits blunt the notch and redistribute the notch tip concentration stresses, achieving the delay of the tensile failure of 0° fibers. An optical micrograph of the pure tension specimens loaded to the 0.85·F_p_ is shown on Figure 6a. In the cross-ply basalt fiber composites, we can find cracks predominantly within the fiber bundles (intra-bundle). This indicates the separation of elementary fibers due to the breakdown of epoxy adhesion between them. This cracking is seen to be evenly distributed among fiber bundles. In the fiber bundles perpendicular to the loading direction, intra-bundle cracks are more frequent than in longitudinal direction, and are mainly constrained inside the bundle. Some cracks that originate from the fiber bundle manage to progress through the matrix until they reach the longitudinal fiber in the form of a transverse crack. Significant tensile stresses cause the formation of cracks, which are predominantly characterized by the opposite movement of the crack sides and consequently lead to the generation of tensile mode AE signals. The increasing load led to the steady growth and merging of intra-bundle cracks and the formation of cracks around fiber bundles, as well as along the fiber–matrix interface, as shown on Figure 6b, for a specimen reaching peak load. Circum-bundle cracks debond the fiber bundle from the matrix. The accumulation of damage lowers the bearing capability of the composite and leaves behind uncracked 0° fibers that bridge the crack, providing resistance to opening the crack. Increased load on fibers leads to the pulling out and cracking of fibers. 

As shown on Figure 5, the failure of the 0° loading specimen occurs slightly above or below the notch, rather than directly at it. This is because the failure of the 0/90 cross-ply begins at the point where longitudinal fibers are cut to manufacture the notch. This area experiences maximum strains due to the notch effect, resulting in strain release. Stress is then relaxed in the regions above and below the notch, leading to higher stress and strain concentration near the notch. In this instance, strain failure occurred at the cut fibers above the notch.

**Figure 7:**
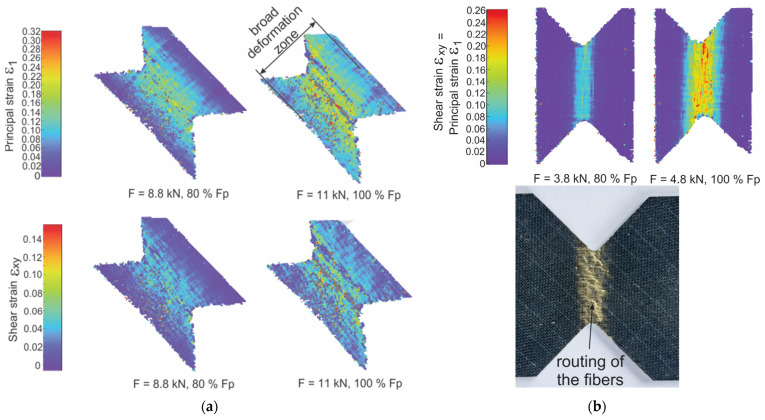
(**a**) 45° and (**b**) 90° loading DIC strain field for principal strain ε_1_ and shear strain ε_xy_, with a macrograph of the 90° sample after failure.

**Figure 8:**
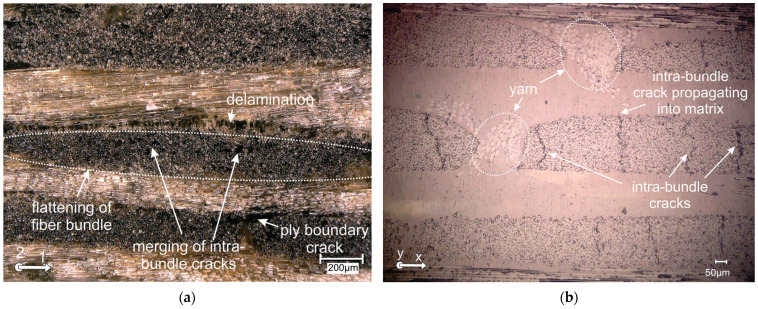
(**a**) 45° loading composite after reaching F_p_; and (**b**) 90° loading composite after 0.85·F_p_.

45° loading of specimens introduces shear stress, and at the same time reduced tensile stresses compared to pure tension. This leads to achieving the maximum total displacement during loading, which is associated with a broad deformation zone (Figure 7a). During 45° loading, laminates also demonstrate intra-bundle cracks, as shown on Figure 8a. These spread over the bundle and combine with each other, which causes the formation of a scattered structure of cracks in the bundle. The cracks originating from the fiber bundle extend into ply boundary cracks (inter-ply cracking). They combine with the cracks of the adjacent bundle and extend to the formation of delamination.

The micrograph on Figure 8b shows a cross-section of the 0.85·F_p_ simple shear specimen. We can notice several through bundle cracks parallel with a loading direction that is primarily constrained inside the bundles. These through bundle cracks, which are characterized by the parallel movement of crack surfaces, could represent the important source of shear mode AE signals. It is rare to encounter a visible phenomenon of intra-bundle crack propagating into matrix. The micrograph also shows yarn fibers in the cross-section, used to stitch together layers in biaxial fabrics. When increasing the load, intra-bundle cracks connect with fiber-matrix cracks. The weakening of the load-bearing capacity of the laminate leads to critical failure modes during shear, i.e., delamination of adjacent plies and fiber breakage. During biaxial and shear loading, fiber rotation occurs in the gauge area. Fibers along the 1 axis tend to lengthen, inducing a tensile stress. Figure 7 is showing DIC principal strain and a shear strain field when near the maximum loading of butterfly specimens. The orientation of the fibers in the direction of maximum load can be noticed on the macrograph of the simple shear loaded sample (Figure 7b). This routing of the fibers orientated along 1 axis coincides with a pronounced increase in failure displacement. The occurrence of fiber rotation during loading and subsequent deformation has a significant effect on the non-linear behaviour of composite laminates [42,43]. During the shear loading, we can expect fibers in the specimen longitudinal direction to rotate, following the movement of the clamps that introduces rigid body rotation. With the increase in loading towards the F_p_, the specimen also undertakes local tension as well as shear, that, apart from just shear mode, also introduces tensile mode AE signals. The specimens in our tests were loaded a little above the F_p_ and, therefore, we did not have a complete separation of the specimens. All specimens failed from the notch in their gauge or near-gauge section. In a case of 0° loading, this failure occurred in a catastrophic manner, while in the case of angled loading, the failure was accompanied by a slow decay of load force.

Described damage modes for different loading cases has different AE signatures depending on the mode of the crack. The tensile mode crack with opposite movement of the crack sides results in AE signals with a short rise time (RT) and high frequency (AF), while the shear types of cracks usually result in longer waveforms, with lower frequency and longer rise time. This leads to the transmission of the larger part of the energy in the form of slower shear waves; therefore, the maximum peak of the waveform is delayed compared to the onset of the initial longitudinal arrivals.

**Figure 9:**
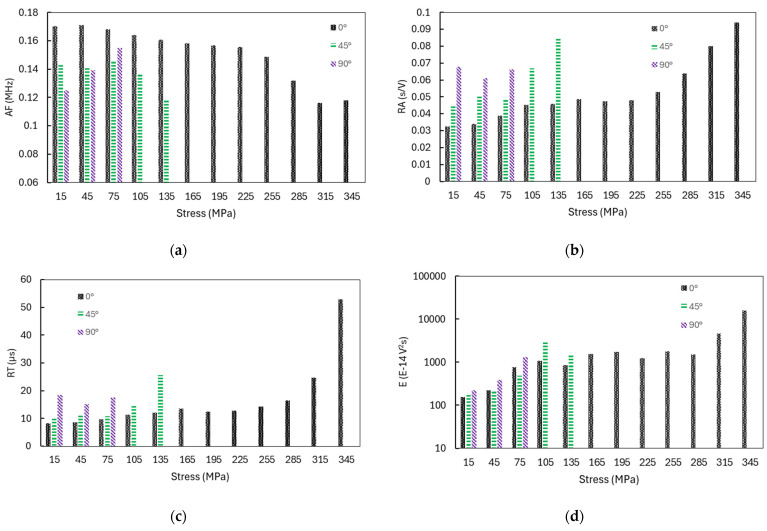
Characteristic AE parameters with Stress (σ_y_ for 0° and 45° loading and τ_xy_ for 90° loading) for (**a**) AF; (**b**) RA; (**c**) RT; and (**d**) E.

The average values of the AE parameters in relation to the loading stress σ_y_ for 0° and 45° loading, and in relation to τ_xy_ for 90° loading, are shown on Figure 9. Abscissa values are the mean values of a 30 MPa large stress interval. Acquired AE signals during an early damage state, in the case of the predominant tensile mode of loading, exhibit higher AF values and lower RA values. With the increase towards final failure, the AF decreases and RA increases. However, different parameter changes can be observed with pure shear loading. AF increases as final failure approaches, which can relate to fiber rotation and the occurrence of tensile cracks in the specimen, while RA values do not show a pronounced trend of increasing as final failure approaches. Shear type cracks with pure shear leads to higher RT values, as expected compared to increased tensile loading connected with tensile mode cracks. Similarly, RA is higher in the case of 45° and 0° loading, while AF is lower than that of tensile loading. The increase in the E with increased stresses can be linked with the emergence of delamination and fiber breaks in a laminate. The value of RT is higher in the early loading phase in simple shear than in the case of pure tensile loading, while, with increasing load towards peak force F_p_, RT values increase significantly in loading cases where the tensile stress is higher, and becomes much higher than in shear loading.

**Figure 10:**
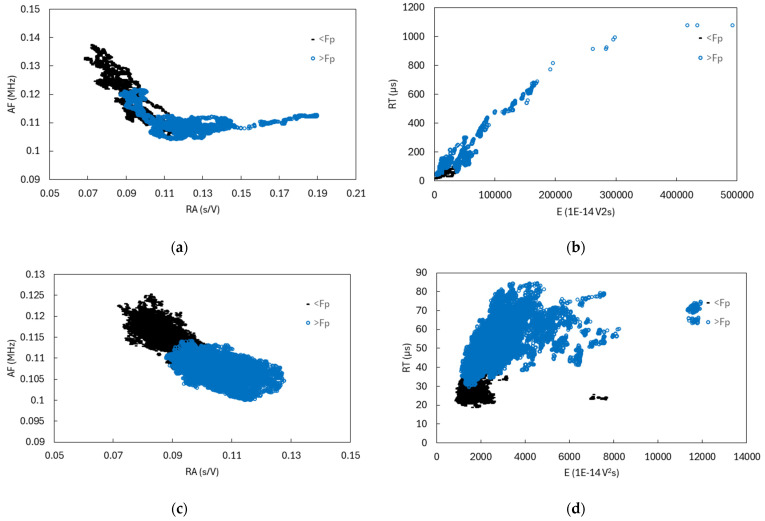

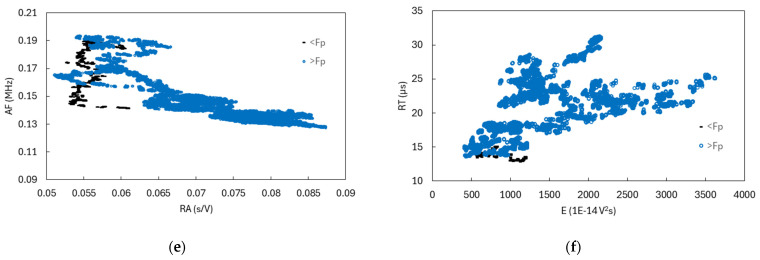
Characteristic AE parameters during the F_p_ transition for 0° (**a**) and (**b**); 45° (**c**) and (**d**); and 90° loading (**e**,**f**).

The passage of F_p_ in the case of different loading modes is shown on Figure 10. The AE signal parameters are presented in the range of 4% before, and 4% after, passing Fp. A narrow F window was chosen that would enable timely AE detection of the maximum load transition. Higher stress levels in 0° samples influence the more energetically pronounced events in the composite, and, consequently, higher energy of AE signals. By directing towards simple shear, the energy of the captured signals decreases, similar to the parameters RT and RA. All three cases of loading show an increase in the values of RA, E and RT after the passage of F_p_. When we get very close to the F_p_ of the 0° FRP sample, a pronounced deformation zone with high strain values is formed above the notch of the sample, which indicates the location of the macroscopic crack. The splitting, transverse cracking and delamination reduces the local stress concentration at the notch tip in the load-bearing direction by redistributing the stress away from the notch, and this delays the onset of a catastrophic tensile fracture of the longitudinal fibers. The crack-blunting effect that strengthens the tensile specimen is connected with the significantly increased number of detected AE hits and increased RA values. As the loading continues, the crack grows towards the inside of the sample, where the fibers are bridging the crack with fiber pull outs. We can detect a marked increase in THE E and RT of the acquired signals. Crack growth is attributed to the reduction of the cross-section of the sample, due to the increase in the crack and the consequent reduction in the number of pulled out fibers.

## 4. Conclusions

In this study, the mechanical properties and the progression of damage of cross-ply basalt fiber composites are investigated under different loading conditions. A customized Arcan rig for simultaneous AE monitoring was developed to apply quasi-isotropic shear, combined tensile and shear forces and pure tensile stresses to specimens with a central notch. DIC technology was used to acquire strain measurements. The analysis includes a detailed investigation of the competing damage mechanisms that lead to a decrease in stress concentration in specific areas. By analyzing different AE signal indicators, the research identifies different damage processes. The experimental results illustrate the relationship between the average acoustic emission (AE) parameters and the applied loading stress at different loading orientations: 0°, 45° and 90°. In the early stages of damage under predominantly tensile loading, the observed AE signals showed increased AF magnitudes together with decreased RA values. This correlates with splitting in the pronounced tows predominantly connected with intra-bundle cracks, with the opposite movement of the crack sides that generate tensile mode AE signals. As the material approaches ultimate failure, there is a significant decrease in AF and an increase in RA values that is characteristic for tension mode crack formation and evolution. With shear stress, however, the behaviour of these parameters shifts. Lower values of AF in the initial phase of loading can be predominantly attributed to the formation of shear intra-bundle cracks that appear in fibers oriented in the loading direction. The AF tends to increase as the loading approaches its peak value. This correlates with the occurrence of more pronounced fiber rotation and the consequent local appearance of tension mode cracks. As expected, shear stresses leading to shear type crack result in higher RT values compared to increased tensile stresses. The characteristic AE parameters during the F_p_ transition are also presented in a narrow F window, which allows the timely AE detection of the maximum load transition. In all three loading cases we can observe a significant increase in the values of RA, E and RT after the transition of F_p_. In the case of pure tension and tension–shear combination, the AF value decreases at the F_p_ transition, while for pure shear there is no significant decrease in AF.

The integration of DIC and AE monitoring improves the understanding of both mechanical behaviour and failure processes in cross-ply basalt fiber composites. This comprehensive approach provides important insights for future performance monitoring and the possibility of structural reliability in a range of engineering applications. The correlations of AE signals with dominant stresses in composites could offer possibilities for the predictions of the stress/damage behaviour in real engineering applications. Ongoing research is also being conducted to provide an advanced damage characterisation of similar bio-epoxy composites in low cycle fatigue experiments. These data will be used as input for fatigue damage characterisation.

## Figures and Tables

**Table 1:** Average peak loads and strengths of notched specimens.

Loading Angle α (°)	Peak Load P (kN)	Tensile Strength σ (MPa)	Shear Strength τ (MPa)
0	23.4	345	0
45	11.9	124	124
90	4.7	0	69

## Data Availability

Data is contained within the article.
